# Impact of air pollution on healthcare utilization in patients with bronchiectasis

**DOI:** 10.3389/fmed.2023.1233516

**Published:** 2023-10-11

**Authors:** Hyun Lee, Sang Hyuk Kim, Sun-Kyung Lee, Hayoung Choi, Sung Jun Chung, Dong Won Park, Tai Sun Park, Ji-Yong Moon, Tae-Hyung Kim, Sang-Heon Kim, Jang Won Sohn, Ho Joo Yoon

**Affiliations:** ^1^Division of Pulmonary Medicine and Allergy, Department of Internal Medicine, Hanyang University College of Medicine, Seoul, Republic of Korea; ^2^Division of Pulmonary, Allergy, and Critical Care Medicine, Department of Internal Medicine, Dongguk University Gyeongju Hospital, Dongguk University College of Medicine, Gyeongju, Republic of Korea; ^3^Department of Mathematics, College of Natural Sciences, Hanyang University, Seoul, Republic of Korea; ^4^Division of Pulmonary, Allergy, and Critical Care Medicine, Department of Internal Medicine, Hallym University Kangnam Sacred Heart Hospital, Hallym University College of Medicine, Seoul, Republic of Korea

**Keywords:** bronchiectasis, public health, environmental pollution, air pollution, particulate matter

## Abstract

**Introduction:**

Air pollutants are increasingly recognized to affect long-term outcomes in patients with bronchiectasis. We aimed to figure out the association between air pollutants and the risk of healthcare utilization in patients with bronchiectasis.

**Methods:**

Data for 1,029 subjects with bronchiectasis in Seoul were extracted. The air pollutants included particulate matter of 10 μm or less in diameter (PM_10_), particulate matter of 2.5 μm or less in diameter (PM_2.5_), sulfur dioxide (SO_2_), carbon monoxide (CO), ozone (O_3_), and nitrogen dioxide (NO_2_). The outcome was all-cause healthcare uses, defined as outpatient visit, emergency department visit, or hospitalization. The concentration–response curves between each air pollutant and relative risks for healthcare utilization were obtained.

**Results:**

There were significant correlations between air pollutant concentrations and the risk of healthcare utilization, particularly for PM_10_, NO_2_, SO_2_, and CO. This risk was observed even at concentrations below the recommended safe thresholds for the general population. The slopes for the association between PM_10_ and NO_2_ and the risk of healthcare use showed a logarithmic growth pattern, with the steepest increase up to 30 μg/m^3^ and 0.030 parts per million (ppm), respectively. The curves for SO_2_ and CO showed an inverted U-shaped pattern, with a peak at 0.0045 ppm and a slow upward curve, respectively. No specific trends were observed for PM_2.5_ and O_3_ and the risk of healthcare use.

**Discussion:**

Increased concentrations of PM_10_, NO_2_, SO_2_, and CO were associated with increased healthcare utilization in patients with bronchiectasis. For patients with bronchiectasis, there were no safety thresholds for those air pollutants, and even low levels of air pollutant exposure can negatively impact bronchiectasis outcomes.

## Introduction

Non-cystic fibrosis bronchiectasis (hereafter referred to as bronchiectasis) is a chronic lung disease characterized by abnormal and permanent dilation of bronchi and respiratory symptoms (1). Once considered an orphan disease, the prevalence and disease burden of bronchiectasis have also been increasing worldwide (2).

In bronchiectasis management, prevention of exacerbation is key to reducing the disease burden (3). Bronchiectasis exacerbation is generally triggered by respiratory infection, but air pollution has been shown to increase the risk of bronchiectasis exacerbation (4, 5). For example, higher levels and acute fluctuations of air pollution were associated with an increased risk of bronchiectasis exacerbation (5). A recent study from China found that air pollution increased the risk of hospital admission in patients with bronchiectasis (6).

Air quality guidelines have been announced in many countries, including Korea, to protect people from the hazardous effects of air pollution (7). These guidelines establish safe thresholds for each air pollutant. However, it is not clear whether these safe thresholds are applicable to patients with bronchiectasis, as previous studies have primarily focused on the acute fluctuation of air pollutants and have not assessed safe thresholds of air pollutants for patients with bronchiectasis.

Therefore, this study aims to investigate the association between air pollutants and healthcare utilization in patients with bronchiectasis, with a focus on determining the safe thresholds for air pollutants in this population.

## Materials and methods

### Study population

The data source was the 2017 Health Insurance Review and Assessment Service, National Patient Sample (HIRA-NPS), which is nationally representative and open to the public for research purposes (8). The HIRA-NPS data are cross-sectional and consists of health insurance claim records accrued in the year. The database includes approximately 1,400,000 individuals each year, drawn from 3% stratified random sampling by age and sex of the population with claims records during the year. It also provides information on healthcare costs, consisting of payers’ amounts and patients’ out-of-pocket costs. Korea has a government-run mandatory national health security system; 97% of the population is enrolled in National Health Insurance and 3% in Medical Aid Programs (2, 9).

**Figure 1:**
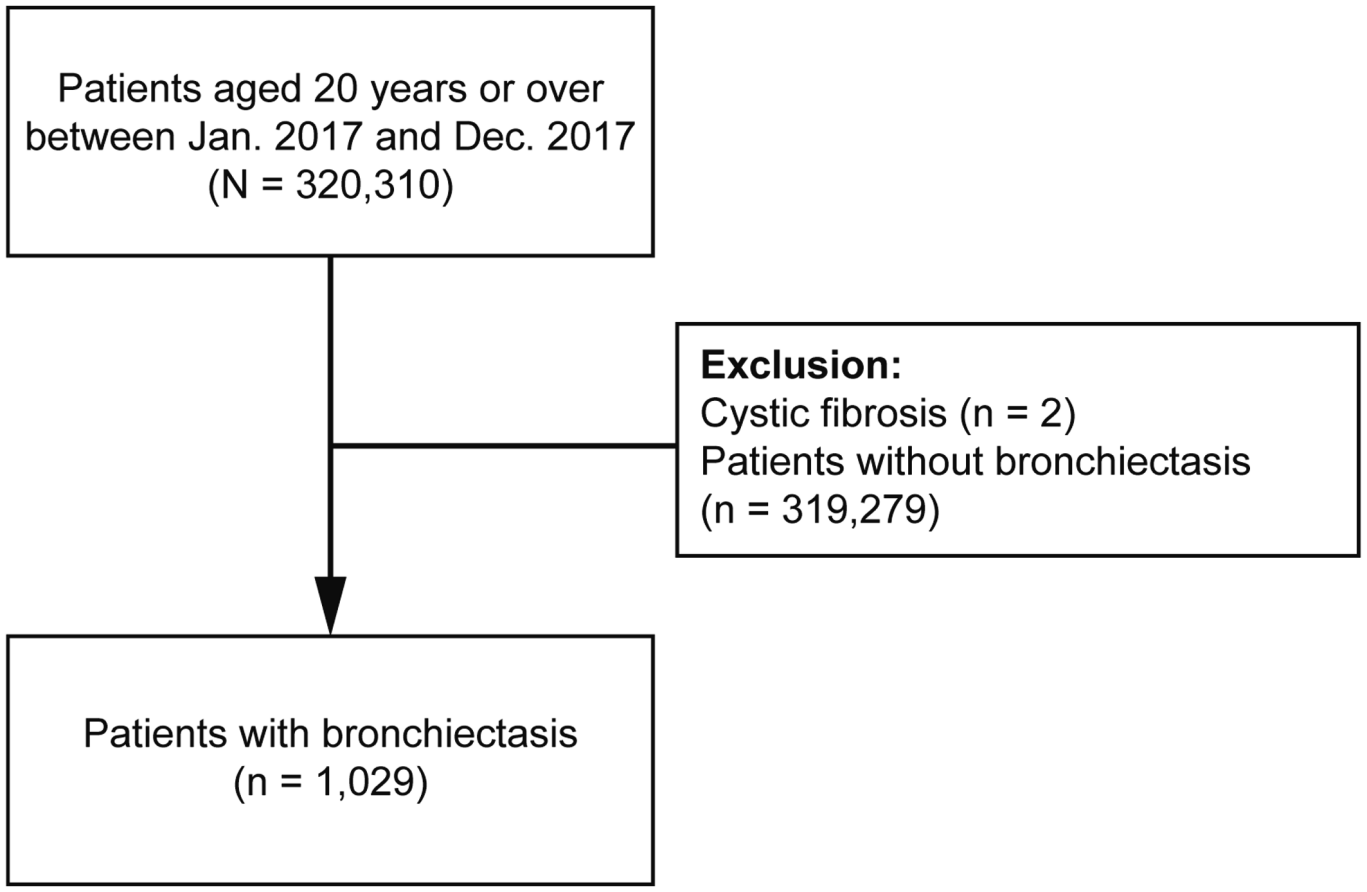
Flow chart of the study population.

This study initially included all patients who were ≥20 years and used hospitals in Seoul, Korea, between 1 January 2017 and 31 December 2017 (*n* = 320,310). We restricted the patients to those who lived in Seoul because it allowed us to acquire more accurate air pollution data. We excluded two patients diagnosed with cystic fibrosis and 319,279 patients without bronchiectasis. Finally, 1,029 patients with bronchiectasis were included (Figure 1).

### Measurement: bronchiectasis and comorbidities

Bronchiectasis was defined by at least one claim under the 10th revision of the International Statistical Classification of Diseases and Related Health Problems (ICD-10) code J47 excluding E84 (cystic fibrosis) during the study period (2).

Comorbidities were defined using the following ICD-10 diagnosis codes: angina pectoris (I20), myocardial infarction (I21–I22, or I25.2), heart failure (I43, I50, I09.9, I11.0, I13.0, I13.2, I25.5, I42.0, I42.5–I42.9, or P29.0), peripheral vascular disease (I70–I71, I73.1, I73.8, I73.9, I77.1, I79.0, I79.2, K55.1, K55.8, K55.9, Z95.8, or Z95.9), cerebrovascular disease (G45–G46, I60–I69, or H34.0), dementia (F00–F03, F05.1, G30, or G31.1), asthma (J45–J46), chronic obstructive pulmonary disease (COPD) [J42–J44 except J43.0 (unilateral emphysema)], connective tissue disease (M05, M06, M31.5, M32–M34, M35.1, M35.3, or M36.0), peptic ulcer disease (K25–K28), liver disease (K70.3, K71.7, K73, K74.3–K74.6, K72.1, K72.9, K76.6, or K76.7), diabetes mellitus (DM) without complication (E10–E14), hemiplegia (G04.1, G11.4, G80.1, G80.2, G81–G82, G83.0–G83.4, or G83.9), and cancers (C00–C97) (2, 10). Cardiovascular disease was defined as angina pectoris, myocardial infarction, or heart failure. The Charlson comorbidity index (CCI) was calculated as previously reported (11).

### Measurement: meteorological and air pollution data

Meteorological data for Seoul (latitude, 37.57° N) were obtained from the database of the Korea Meteorological Administration: 24 h mean temperature, relative humidity, and sea-level air pressure. Data on outdoor air pollution were obtained from Air Korea, the open database from the Ministry of the Environment (Korean government) (12). Daily mean concentrations were recorded at 25 observatory stations in Seoul during the study period (13, 14).

### Air pollutants and air quality standards

The air pollutants included particulate matter of 10 μm or less in diameter (PM_10_), particulate matter of 2.5 μm or less in diameter (PM_2.5_), nitrogen dioxide (NO_2_), sulfur dioxide (SO_2_), ozone (O_3_), and carbon monoxide (CO). The air quality standards of Korea and the World Health Organization (WHO) are provided in Supplementary Table S1 (12, 15).

### Study outcome

The main outcome of this study was all-cause healthcare uses in 2017, including outpatient department visits, emergency room visits, or hospitalization.

### Statistical analysis

Data are presented as frequencies (percentages) for categorical variables. We evaluated the relative risk (RR) for increased healthcare utilization according to air pollution concentration using concentration–response curves with the lowest pollutant level as the reference. The generalized additive model (GAM) based on Poisson distribution was used to examine the association between air pollution and healthcare use. The smoothing splines with degrees of freedom included in GAM control the variations in the generalized linear model (GLM) equation. Degrees of freedom are determined by the smallest Akaike’s information criterion (AIC) value, which suggests the best statistical model fits (16). We used the natural cubic splines to adjust for nonlinear confounding variables of daily average temperature and seasonality with seven and four degrees of freedom, respectively.

Additionally, we included the special daily trends using a dummy variable to account for the effects of weekends and holidays on healthcare use. The daily average relative humidity was corrected by applying a linear function. The multivariable analysis, which includes two or more pollutants, was not conducted because of the significant correlation among air pollutants (Table 1). All statistical analyses were performed using SAS 9.4 (SAS Institute, Cary, NC, United States) and R 3.6.1 (R Foundation for Statistical Computing, Vienna, Austria).

**Table 1:** Spearman correlation coefficients between concentrations of air pollutants in Seoul, Korea in 2017.

	PM_10_	PM_2.5_	NO_2_	SO_2_	O_3_	CO	Temperature	Relative humidity
PM_10_	1.000	0.875^**^	0.558^**^	0.706^**^	0.119^*^	0.609^**^	−0.274^**^	−0.141^**^
PM_2.5_		1.000	0.658^**^	0.708^**^	0.006	0.752^**^	−0.229^**^	0.062
NO_2_			1.000	0.677^**^	−0.348^**^	0.845^**^	−0.218^**^	−0.012
SO_2_				1.000	−0.124^*^	0.665^**^	−0.413^**^	−0.221^**^
O_3_					1.000	−0.467^**^	0.512^**^	−0.121^*^
CO						1.000	−0.414^**^	0.070
Temperature							1.000	0.426^**^
Relative humidity								1.000

^*^ and ^**^ indicates *p* < 0.05 and *p* < 0.01, respectively. PM_10_, particulate matter of 10 μm or less in diameter; PM_2.5_, particulate matter of 2.5 μm or less in diameter; NO_2_, nitrogen dioxide; SO_2_, sulfur dioxide; O_3_, ozone; CO, carbon monoxide.

## Results

### Population, air pollutants, and metrological data

The baseline characteristics of the study population are presented in Table 2. The majority of patients were older than 60 years (67.9%), female (60.5%), and had self-employed health insurance (93.2%). Nearly three-fourths of patients had a CCI score ≥2 (76.2%). The most common comorbid condition was dyslipidemia (52.0%), followed by asthma (36.6%), COPD (34.5%), diabetes mellitus (25.7%), cardiovascular disease (15.2%), hypertension (14.4%), and cancers (10.4%). A total of 4.8% of patients had a history of pulmonary tuberculosis. Summary statistics for air pollutants and meteorological data during the study period are described in Supplementary Table S2.

**Table 2:** Baseline characteristics of the study population.

	Total (*n* = 1,029)
**Age, years**	
20–29	8 (0.8)
30–39	22 (2.1)
40–49	61 (5.9)
50–59	240 (23.3)
60–69	369 (35.9)
≥70	329 (32.0)
**Sex**	
Male	406 (39.5)
Female	623 (60.5)
**Type of insurance**	
Self-employed health insurance	959 (93.2)
Employee health insurance	59 (5.7)
Medical aid	11 (1.1)
**CCI score**	
1	245 (23.8)
≥2	784 (76.2)
**Comorbidities**	
COPD	355 (34.5)
Asthma	377 (36.6)
A history of pulmonary tuberculosis	49 (4.8)
Cancer	107 (10.4)
Hypertension	426 (14.4)
Diabetes mellitus	264 (25.7)
Dyslipidemia	535 (52.0)
Cardiovascular disease	156 (15.2)

Values are presented as numbers (%). CCI, Charlson comorbidity index; COPD, chronic obstructive pulmonary disease.

### Correlation between concentrations of air pollutants

There were significant correlations between six pollutants, except PM_2.5_ and O_3_ (Table 1). Particularly, strong correlations were found between the following pollutants: PM_10_–PM_2.5_, PM_10_–SO_2_, PM_2.5_–SO_2_, PM_2.5_–CO, and NO_2_–CO. Most of the associations between air pollutants showed positive correlations, except for O_3_, which showed a negative correlation with NO_2_, SO_2_, and CO. Temperature was negatively correlated with all air pollutants, except for O_3_.

### Effects of air pollution on healthcare utilization

**Figure 2:**
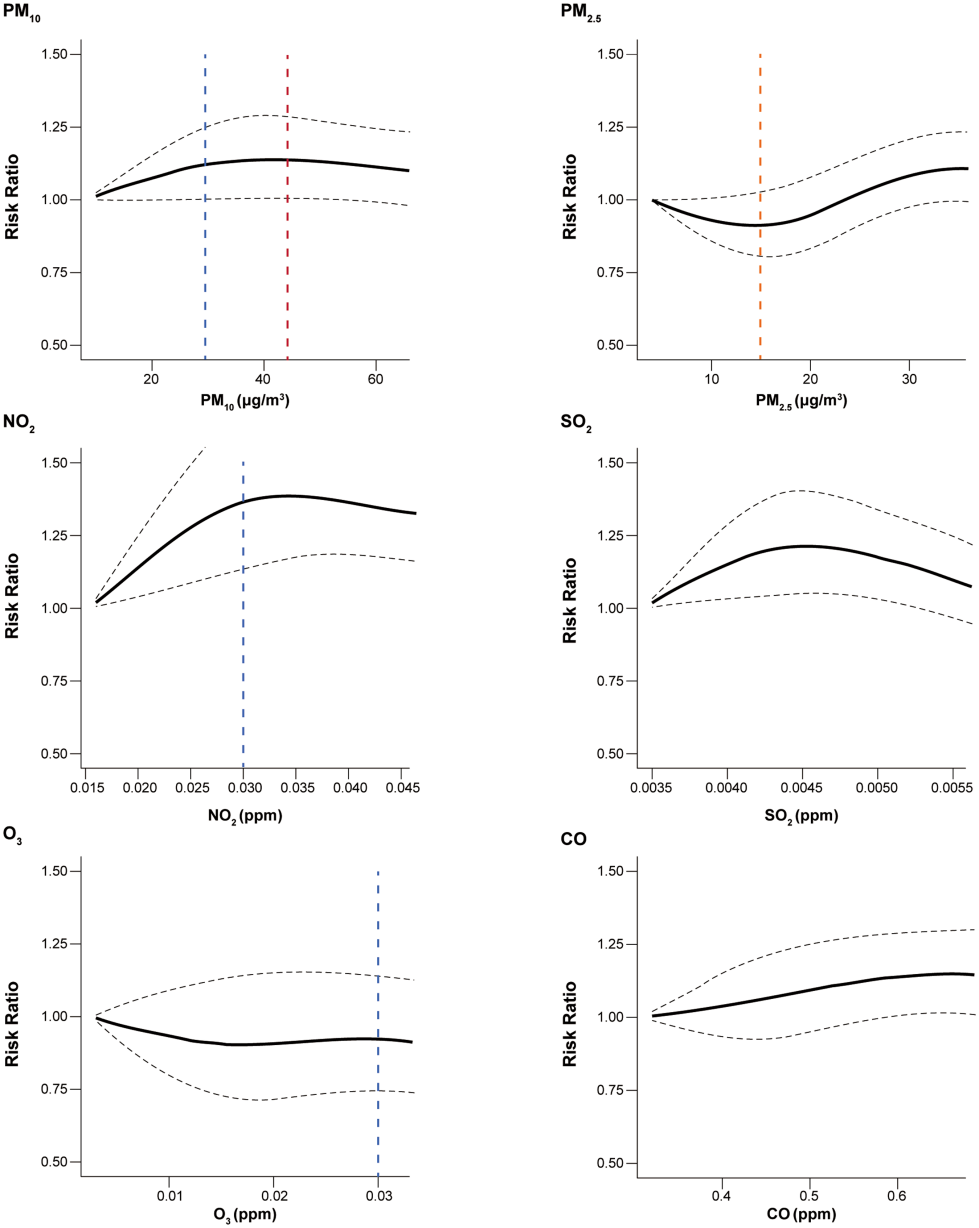
Concentration–response curves for the association between all-cause healthcare utilization and air pollutants. Black dashed line indicates 95% confidence interval. Blue, red, orange vertical dashed lines indicate, the Korean, WHO, and the shared threshold for good air quality, respectively. The safe thresholds for SO_2_ and CO were higher than observed values. PM_10_, particulate matter of 10 μm or less in diameter; PM_2.5_, particulate matter of 2.5 μm or less in diameter; NO_2_, nitrogen dioxide; SO_2_, sulfur dioxide; O_3_, ozone; CO, carbon monoxide; WHO, World Health Organization.

Figure 2 depicts concentration–response curves between the air pollutants and RR for healthcare utilization. There was a significant increase in RRs for healthcare utilization with increasing air pollutant concentration for PM_10_, NO_2_, SO_2_, and CO. Notably, NO_2_, SO_2_, and CO were associated with increased risk of healthcare utilization even below the recommended safe thresholds. The slopes for the association between PM_10_ and NO_2_ and the risk of healthcare utilization showed a logarithmic growth, with the steepest increase up to 30 μg/m^3^ and 0.030 parts per million (ppm), respectively. The curves for the association between SO_2_ and CO and healthcare utilization showed an inverted U shape with a peak at 0.0045 ppm and a slow upward curve, respectively. However, no specific trends were observed for the association between PM_2.5_ and O_3_ and the risk of healthcare utilization.

## Discussion

In this population-based cohort study, PM_10_, NO_2_, SO_2_, and CO were associated with increased healthcare utilization in patients with bronchiectasis. Also, we found that healthcare utilization increased even levels below the safe thresholds for those air pollutants in a concentration-dependent manner. These results suggest that there are no safe thresholds for air pollutants in patients with bronchiectasis and that even low levels of exposure can negatively impact bronchiectasis.

Our findings that air pollution increases the risk of healthcare use in patients with bronchiectasis support previous studies (5, 6, 17). The potentially deleterious effects of air pollution on the prognosis of bronchiectasis were first reported by Goeminne and his colleagues (17). In their study, patients with bronchiectasis who lived near major roads were more likely to die than those who did not. In a subsequent study, the authors found that short-term changes in air pollution are associated with an increased risk of exacerbation of bronchiectasis regardless of subtype or severity of bronchiectasis (5). Recently, a population-based study in southern China found that the risk of hospitalization for bronchiectasis increased by 3.8%–6.7% as the interquartile range of air pollution increased (6).

Although there is limited scientific evidence, some plausible explanations exist for the association between air pollution and increased healthcare utilization in patients with bronchiectasis. First, the direct effects of air pollutants may cause bronchial constriction. High levels of NO_2_ and SO_2_ are associated with the irritant response of the airway (18). Second, air pollutants may cause systemic inflammation associated with bronchiectasis exacerbation. Exposure to particulate matter can activate a variety of cytokines [e.g., tumor necrosis factor (TNF)-α, interleukin (IL)-6, and IL-13] involved in the pathogenesis of chronic airway inflammation (19). Third, lung microbiome status might be altered due to air pollution. Air pollution can make patients with bronchiectasis more prone to respiratory infection (20), which may lead to dysbiosis in the lower airway. Previous studies have reported a link between air pollution and increased risk of respiratory infections, including SARS-CoV-2 and influenza virus (21). These respiratory infections can disrupt the normal balance of microbes in the lungs, making it easier for other infections to take hold (22). Since bacterial colonization is an important factor in bronchiectasis management, disrupting the balance of the lung microbiome may affect bronchiectasis exacerbation (23).

In our study, significant correlations were found between air pollutants, with some strong relationships. This suggests the possibility that air pollutants work together to worsen bronchiectasis. It has been reported that PM_2.5_ exhibits a synergistic effect when combined with other pollutants; for example, simultaneous exposure to PM_2.5_ and O_3_ aggravated bronchial asthma in the animal study (24). Nevertheless, there has been no established comprehensive integrated air pollution index, and most countries only provide individual air pollutant levels. The complex interplay between air pollutants makes it difficult to predict how they affect individual patients, but it seems clear they can play a role in the worsening of bronchiectasis. Therefore, further studies should focus on developing an integrated air pollution model aimed at assessing its effect on bronchiectasis.

We confirmed that there are no safety thresholds for air pollutants, particularly for PM_10_, NO_2_, SO_2_, and CO in patients with bronchiectasis. The significant association between these air pollutants and increased risks for healthcare utilization in patients with bronchiectasis was observed even below the good air quality values set by the Korean and WHO guidelines (15). Several countries and organizations still provide thresholds for air pollution based on the results of studies evaluating the effect of air pollutants on mortality (25, 26), which were conducted in a single region or country that did not focus on the thresholds of air pollutants. A meta-analysis from the Multi-City Multi-Country (MCC) data, encompassing 652 cities in 24 countries/regions, revealed that PM_10_ and PM_2.5_ increased the mortality without identifiable thresholds (27). Moreover, a recent study using Medicaid data demonstrated that air pollutant exposures were associated with increased risk of asthma hospitalization, below the level of U.S. standard (28). Considering these outcomes and our study results, it is suggested that there is no safety threshold for air pollution in the general population as well as patients with bronchiectasis.

Notably, our study showed that the increase in healthcare utilization risk per unit increase in PM_10_ and NO_2_ was more pronounced at below the safety thresholds, demonstrating a logarithmic growth pattern. Previous studies showed similar results that per unit increase of PM_10_ and PM_2.5_ at lower concentrations more substantially increased mortality and cardiovascular hospitalization compared to the increase at higher concentrations (27, 29). While the mechanism for these observations remains unclear, our study contributes additional evidence to the previous studies, urging efforts to reduce air pollution irrespective of prescribed safety thresholds recommended to the general population. Current policies aimed at maintaining concentrations of air pollution just below specific levels should be reconsidered. Through continuous improvements in air pollution, patients with bronchiectasis will be able to achieve improved health outcomes.

We should address some limitations of our study. First, we used ICD-10 codes for the definition of bronchiectasis. There might have been under or over-diagnosis in all studies using claim data. Second, this study was conducted only in 1 year. Therefore, comprehensive analysis, such as lagged day model and moving average, was unsuitable for our analysis. Third, we could not conduct a sensitivity analysis due to a small number of patients and the nature of claim data resulting in an outcome limited to all-cause healthcare utilization. Fourth, only air pollution data in the residential area were used without consideration of that in the workplace. If study subjects work outside of Seoul, their exposure to air pollution may be different. However, since most of the patients were elderly people aged 60 or older who were likely to have retired from work, the effect of this factor may not be significant. Fifth, previously known risk factors for exacerbation of bronchiectasis, such as lung function, microbial colonization, and bronchiectasis severity index, could not be evaluated due to a lack of information. Sixth, due to limited healthcare utilization details, routine hospital visits might be included as healthcare utilization. However, given their regularity, their influence on the study’s outcomes is likely minimal. Finally, since data were obtained from a major city in a single country, caution is needed in generalizing our results.

In conclusion, PM_10_, NO_2_, SO_2_, and CO were associated with increased healthcare utilization in patients with bronchiectasis, even below the recommended safe thresholds.

## Data availability statement

The datasets presented in this article are not readily available because the HIRA-NPS database is for use only by designated persons. Requests to access the datasets should be directed to http://opendata.hira.or.kr.

## Ethics statement

The studies involving humans were approved by Institutional Review Board (IRB) of Hanyang University Hospital (application no. HYUH 2022-08-043). The studies were conducted in accordance with the local legislation and institutional requirements. The ethics committee/institutional review board waived the requirement of written informed consent for participation from the participants or the participants’ legal guardians/next of kin because the HIRA-NPS database was constructed after anonymization.

## Author contributions

HY: study conception and design. S-KL: data analysis. HL and SK: data interpretation and manuscript writing. HC, SC, DP, TP, J-YM, T-HK, S-HK, and JS: revision of manuscript and contribution of intellectual content. All authors contributed to the article and approved the submitted version.
