# Supplementary material for: Impact of air pollution on healthcare utilization in patients with bronchiectasis
Source: Front Med (Lausanne). 2023 Oct 11;10:1233516. doi: 10.3389/fmed.2023.1233516 (PMC10598766; doi:10.3389/fmed.2023.1233516)
Supplement: Supplementary file 1 [file Table_1.DOCX]

**Supplemental Table 1.** Recommended air pollutant levels in air quality guidelines.

|  | Korean standard | | | | WHO guideline (2021) |
| --- | --- | --- | --- | --- | --- |
|  | Good | Moderate | Bad | Very bad |  |
| PM_10_ (μg/m^3^) | 30^*^ | 80^*^ | 150^*^ | 600^*^ | 45^*^ |
| PM_2.5_ (μg/m^3^) | 15^*^ | 35^*^ | 75^*^ | 500^*^ | 15^*^ |
| NO_2_ (ppm) | 0.03^‡^ | 0.06^‡^ | 0.2^‡^ | 2^‡^ | 0.1^‡^ |
| SO_2_ (ppm) | 0.02^‡^ | 0.05^‡^ | 0.15^‡^ | 1^‡^ | 0.02^*^ |
| O_3_ (ppm) | 0.03^‡^ | 0.09^‡^ | 0.15^‡^ | 0.6^‡^ | 0.05^†^ |
| CO (ppm) | 2^‡^ | 9^‡^ | 15^‡^ | 50^‡^ | 35^‡^ |

^*^24-hour average concentration. ^†^8-hour average concentration. ^‡^1-hour average concentration.

***Abbreviations***: WHO, World Health Organization; PM_10_, particulate matter of 10 µm or less in diameter; PM_2.5_, particulate matter of 2.5 µm or less in diameter; NO_2_, nitrogen dioxide; SO_2_, sulfur dioxide; O_3_, ozone; CO, carbon monoxide; ppm, parts per million.
