# Supplementary material for: Impact of air pollution on healthcare utilization in patients with bronchiectasis
Source: Front Med (Lausanne). 2023 Oct 11;10:1233516. doi: 10.3389/fmed.2023.1233516 (PMC10598766; doi:10.3389/fmed.2023.1233516)
Supplement: Supplementary file 2 [file Table_2.DOCX]

**Supplemental Table 2.** Summary statistics for ambient air pollutants and meteorological data in Seoul, Korea in 2017

|  |  | Quantiles | | | | | Mean | SD |
| --- | --- | --- | --- | --- | --- | --- | --- | --- |
|  |  | Min | Q1 | Median | Q3 | Max |  |  |
| Air pollutants | PM_10_, µg/m^3^ | 8.0 | 30.9 | 43.8 | 57.8 | 194.8 | 47.3 | 23.6 |
|  | PM_2.5_, µg/m^3^ | 3.9 | 14.7 | 22.0 | 31.4 | 94.8 | 24.7 | 14.2 |
|  | NO_2_, ppm | 0.015 | 0.027 | 0.034 | 0.042 | 0.068 | 0.035 | 0.011 |
|  | SO_2_, ppm | 0.003 | 0.004 | 0.005 | 0.005 | 0.008 | 0.005 | 0.001 |
|  | O_3_, ppm | 0.003 | 0.014 | 0.021 | 0.029 | 0.065 | 0.022 | 0.011 |
|  | CO, ppm | 0.308 | 0.438 | 0.512 | 0.629 | 1.253 | 0.551 | 0.164 |
| Weather | Temperature, °C | -9.4 | 3.0 | 15.1 | 22.6 | 31.4 | 13.1 | 11.0 |
|  | Relative humidity, % | 21.8 | 47.0 | 56.9 | 67.6 | 96.5 | 57.7 | 14.5 |

***Abbreviations***: SD, standard deviation; PM_10_, particulate matter of 10 µm or less in diameter; PM_2.5_, particulate matter of 2.5 µm or less in diameter; NO_2_, nitrogen dioxide; SO_2_, sulfur dioxide; O_3_, ozone; CO, carbon monoxide.
